# Vibration-Controlled Transient Elastography and Controlled Attenuation Parameter for the Diagnosis of Liver Steatosis and Fibrosis in Patients with Nonalcoholic Fatty Liver Disease

**DOI:** 10.3390/diagnostics11050787

**Published:** 2021-04-27

**Authors:** Sebastian Zenovia, Carol Stanciu, Catalin Sfarti, Ana-Maria Singeap, Camelia Cojocariu, Irina Girleanu, Mihaela Dimache, Stefan Chiriac, Cristina Maria Muzica, Robert Nastasa, Laura Huiban, Tudor Cuciureanu, Anca Trifan

**Affiliations:** 1 Department of Gastroenterology, Grigore T. Popa University of Medicine and Pharmacy, 700115 Iasi, Romania; sebastianzenovia20@gmail.com (S.Z.); cvsfarti@gmail.com (C.S.); anamaria.singeap@yahoo.com (A.-M.S.); cameliacojocariu@yahoo.com (C.C.); gilda_iri25@yahoo.com (I.G.); mimidimache@yahoo.com (M.D.); stefannchiriac@yahoo.com (S.C.); lungu.christina@yahoo.com (C.M.M.); robertnastasa948@gmail.com (R.N.); huiban.laura@yahoo.com (L.H.); drcuciureanutudor@gmail.com (T.C.); ancatrifan@yahoo.com (A.T.); 2 St. Spiridon Emergency Hospital, 700115 Iasi, Romania

**Keywords:** NAFLD, fibrosis, steatosis, CAP

## Abstract

Vibration-Controlled Transient Elastography (VCTE) with Controlled Attenuation Parameter (CAP) is a widely used non-invasive technique for concomitant assessment of liver steatosis and fibrosis in patients with nonalcoholic fatty liver disease (NAFLD). We aimed to evaluate the level both of hepatic steatosis and fibrosis as well as the associated risk factors in patients referred to our unit with clinically suspected NAFLD or diagnosed by abdominal ultrasonography. Two hundred four patients were prospectively included in this study and assessed by VCTE with CAP. The final analysis included 181 patients with reliable liver stiffness measurements (LSMs) (53% female, mean age 57.62 ± 11.8 years and BMI 29.48 ± 4.85 kg/m^2^). According to the cut-off values for steatosis grading, there were 10 (5.5%) patients without steatosis (S0), 30 (16.6%) with mild (S1), 45 (24.9%) moderate (S2), and 96 (53%) severe (S3) steatosis. Based on LSM, there were 73 (40.3%) patients without fibrosis (F0), 42 (23.2%) with mild (F1), 32 (17.7%) significant (F2), 19 (10.5%) advanced (F3) fibrosis, and 15 (8.3%) with cirrhosis (F4). In addition, we found an association between several metabolic components and hepatic steatosis and fibrosis. Thus, in the multivariate analysis, higher BMI, fasting plasma glucose, triglycerides, low-density lipoprotein cholesterol, and serum uric were associated with increased CAP. Furthermore, higher serum uric acid and alpha-fetoprotein together with lower platelets count and albumin levels were associated with increased LSM. The assessment of steatosis and fibrosis using VCTE and CAP should be performed in all patients with suspected or previously diagnosed NAFLD in units with available facilities.

## 1. Introduction

Nonalcoholic fatty liver disease (NAFLD) has become a frequent cause of chronic liver disease in our century, affecting one quarter of adults worldwide [1]. The global burden of NAFLD is rising in parallel with increasing rates of obesity, type 2 diabetes mellitus (T2DM), and metabolic syndrome (MeS) [2,3]. NAFLD encompasses two histologically distinct types in individuals without significant alcohol consumption (>30 g/day in men and >20 g/day in women): (1) Nonalcoholic fatty liver (NAFL), considered the “silent” or benign subtype, includes steatosis only, with fat accumulation in liver volume more than 5%, and (2) nonalcoholic steatohepatitis (NASH), the “active” or malignant form, defined as steatosis, hepatocyte ballooning, and inflammation, which is associated with a rapid progression to liver fibrosis, cirrhosis, and, eventually, hepatocellular carcinoma (HCC) [4,5,6]. The prevalence of NASH in NAFLD patients ranges from 10% to 59% among those who underwent liver biopsy (LB) [1]. Although it is estimated that one in three patients with NAFLD has NASH, the true prevalence of NASH is not known, certainly due to the asymptomatic course of the disease, meaning that millions of people worldwide are at risk of cirrhosis [1,5]—a common cause of HCC-related liver transplantation [7].

To date, LB remains the gold standard method for NAFLD diagnosis and activity disease assessment, but routine use of this invasive technique is limited because of the high cost, sampling errors, intra- and inter-observer variability, poor patient acceptance, and a low but real risk of morbidity and mortality; therefore, it cannot be performed for disease diagnosis and monitoring [8,9]. These limitations of LB have led to the development of several new non-invasive methods for liver fibrosis assessment using both biochemical markers (e.g., FIB-4—Fibrosis-4 index, APRI—aspartate aminotransferase to platelet ratio index) and advanced imaging techniques (e.g., VCTE—Vibration-Controlled Transient Elastography, 2D shear wave elastography, acoustic radiation force impulse) in current practice. The most important predictor for poor outcomes, including overall mortality rates, is liver fibrosis stage. Therefore, identifying patients with severe liver fibrosis is mandatory, and this could be achieved by using a non-invasive method such as VCTE, with high acceptability among patients [10]. 

Globally, the first-line widespread imaging test for evaluating hepatic steatosis in individuals with suspected NAFLD is abdominal ultrasonography (US). Despite its practical advantage (low-cost and easy accessibility), US has several limitations including operator dependency and uncertainty for detecting steatosis in those with severe fibrosis, morbid obesity, or in those with less than 20% of liver fat accumulation [11,12,13].

VCTE is a non-invasive method for the evaluation of liver fibrosis, and it has been validated and recommended by guidelines over the years for fibrosis assessment mostly in chronic viral hepatitis [14,15,16], as well as in NAFLD [12,17,18]. Since 2013, the FibroScan^®^ (Echosens, Paris, France) devices have been equipped with controlled-attenuation parameter (CAP), a technique which allows concomitant assessment of hepatic fibrosis and steatosis [19]. Briefly, the physical principle is based on the speed of mechanically generated shear-wave through hepatic volume (more than 100 times than LB core) using pulse-echo ultrasonic acquisitions, which is subsequently converted mathematically into liver stiffness measurement (LSM) using Young’s modulus [20]. Consequently, based on the properties of ultrasonic attenuation of the echo wave in the same hepatic volume due to fat accumulation, CAP mode is considered a surrogate marker for detection and quantification of hepatic steatosis [21,22]. This bedside technique is rapid, painless, and operator-independent, with great repeatability and reproducibility, being widespread nowadays in clinical practice. Likewise, FibroScan^®^ is equipped with two types of probes for adults (M—standard, and XL—for obese subjects), a software of probe selection choosing automatically the type of probe, based on the skin-to-capsule distance which overcomes its prior limitations, leading to a VCTE failure less than 5% [19,23]. Compared to US, CAP quantitatively measures hepatic steatosis with higher sensitivity and specificity, thereby proving an efficient and effective tool alongside VCTE for screening, diagnosis, and follow-up of patients with NAFLD [24].

Herein, the aims of this prospective study were to confirm the diagnosis of NAFLD and measure the degree of the hepatic steatosis as well as the stage of liver fibrosis in patients referred by general practitioners and colleagues in other specialties with clinically suspected or US diagnosed NAFLD, using VCTE and CAP mode. In addition, we aimed to evaluate the risk factors associated with liver steatosis and fibrosis.

## 2. Materials and Methods

### 2.1. Patients

Two hundred four patients with clinically suspected and/or US diagnosed NAFLD, referred to our clinic by general practitioners and colleagues in other specialties, were prospectively enrolled from September 2019 to February 2020 at the Gastroenterology and Hepatology Institute, in Iasi, Romania. The inclusion criteria were (1) age ≥18 years; (2) signed informed consent. Exclusion criteria: (1) excessive daily alcohol consumption (defined as >30 g/day for males and >20 g/day for females); (2) history of other causes of chronic liver disease (primary biliary cirrhosis, hepatotropic viruses or HIV co-infection, Wilson’s disease, hemochromatosis, or autoimmune hepatitis, history of congestive heart failure, drug-induced hepatitis); (3) other causes of hepatic steatosis: steatogenic medication (chemotherapy, corticosteroids, tamoxifen, methotrexate, amiodarone, estrogen); (4) aspartate aminotransferase (AST)/alanine aminotransferase (ALT) ≥10 times the upper limit of normal, or total bilirubin level ≥ 5 mg/dL; (5) pregnant women. The study was performed according to the guidelines of the Declaration of Helsinki and approved by our Institution’s Ethics Committee.

### 2.2. Clinical and Laboratory Assessment

Demographic and clinical details were collected: gender, age, body mass index (BMI), the presence of diabetes (taking anti-diabetic drugs or fasting glucose ≥126 mg/dL), arterial hypertension (antihypertensive drugs use, systolic blood pressure ≥140 mmHg or diastolic blood pressure ≥90 mmHg). Fasting blood tests were collected at baseline: platelet count, aspartate and aminotransferases (ALT, AST), gamma-glutamyl-transferase (GGT), alkaline phosphatase (ALP), total bilirubin (TB), albumin, fasting plasma glucose, ferritin, serum uric acid, serum urea and creatinine, total cholesterol, triglycerides, low-density lipoprotein cholesterol (LDL-c), high-density lipoprotein cholesterol (HDL-c), ferritin, C-reactive protein (CRP), and alpha-fetoprotein (AFP). For each patient, we calculated the most commonly used surrogate serum fibrosis markers using standardized equations (APRI [25], FIB-4 index [26], and NAFLD fibrosis score (NFS) [27]).

### 2.3. VCTE Examination

All patients were examined using FibroScan^®^ 502 Touch (Echosens, Paris, France) by an experienced physician (with hundreds of examinations performed). Patients were examined after an overnight or at least 8-hour fasting, in a lying supine position with the arm in maximal abduction, targeting the right hepatic lobe in one of the intercostal spaces (from 9th to 11th on the midaxillary line). First, the examination was performed with the M probe (standard probe, with a transducer frequency of 3.5 MHz), while the probe switching was made at device indication by real-time probe selection software with the XL probe [(obese-specific probe—using a low ultrasound frequency of 2.5 MHz vs. 3.5 MHz, a large tip diameter (12 mm vs. 9 mm), allowing a greater depth of measurement (35–75 mm vs. 25–65 mm)]. A reliable examination was considered to be if ≥10 valid measurements were obtained with an interquartile range/median (IQR/M) ratio ≤30%. LSM results were expressed in kilopascals (kPa) ranging from 1.5 to 75 kPa, with cut-off staging values as follows: F0 (without), F1 (mild), F2 (significant), F3 (advanced) fibrosis, and F4 (cirrhosis); <5.5, 5.6–7.1, 7.2–9.4, 9.5–12.4, ≥12.5 kPa [28,29]. CAP was expressed in decibels/meter (dB/m) ranging from 100 to 400 dB/m with values indicating S0 (without), S1 (mild), S2 (moderate), S3 (severe) steatosis; <237, 237, 259, 291 dB/m [22]. 

### 2.4. Statistical Analyses

The data were analyzed using IBM SPSS, Version 22.0 (IBM SPSS Inc, Chicago, IL, USA). Statistics for categorical variables are expressed as numbers (percentage). Baseline characteristics and clinical variables were expressed as mean ± standard deviation, if normally distributed, or as median (25th and 75th percentiles), if not normally distributed. Distribution analysis was performed using the Kolmogorov–Smirnov test. An unpaired *t*-test was used for comparison of continuous variables between groups for normally distributed data, and Mann–Whitney or the Kruskal–Wallis test was used to analyze skewed data. One-way ANOVA was considered appropriate to assess the difference in LSM values according to the degree of steatosis. Univariate linear regression was firstly performed to identify the factors that may influence the CAP and LSM values, followed by multivariate linear regression using only the significant factors. Pearson correlation coefficient (r) was used for establishing the association between two variables. A *p*-value of < 0.05 (two-tailed) was considered statistically significant. Only complete data sets were analyzed.

## 3. Results

### 3.1. Patient Characteristics

In this study, 204 patients who were clinically suspected or diagnosed with NAFLD based on US were enrolled and evaluated using VCTE with CAP. A total of 23 (11.3%) patients were excluded (unreliable measurements in 18 of them, and examination failure without measurement values in 5 patients). Finally, 181 patients with reliable acquisitions were included (53% females with a mean age of 57.62 ± 11.8 years), 96 (52.5%) of whom were examined with the M probe and 85 (47.5%) with the XL probe, with a median CAP and LSM values in all measurements of 293 (245.5–339) dB/m and 6.1 (4.8–8.3) kPa, respectively. Obesity, T2DM, and hypertension were present in 87 (48%), 39 (21.5%), and 54 (29.8%) of patients, respectively. Baseline characteristics of all patients with reliable acquisitions included in the final analysis are summarized in Table 1. Out of 181 patients analyzed, 171 (94.5%) had different grades of steatosis. These patients were older (mean age 59.19 ± 11.05 years, *p* < 0.001), with an increased BMI (30.37 ± 4.56, kg/m2, *p* < 0.001), and presented T2DM (*p* < 0.001) and hypertension (*p* < 0.001) in a higher proportion compared to those without steatosis. According to serological parameters, patients with steatosis presented a higher level of fasting plasma glucose (*p* = 0.024), ferritin (*p* = 0.047), CRP (*p* = 0.048), total cholesterol (*p* = 0.031), LDL-c (*p* = 0.009), and serum uric acid (*p* < 0.001), and a low level of HDL-c (*p* = 0.006). Furthermore, they also had increased LSM values (*p* = 0.049), FIB-4 index (*p* = 0.027), NFS (*p* = 0.007), and CAP score (*p* < 0.001) (Table 1).

### 3.2. CAP and LSM Values According to Steatosis Degree and Fibrosis Stage

**Figure 1:**
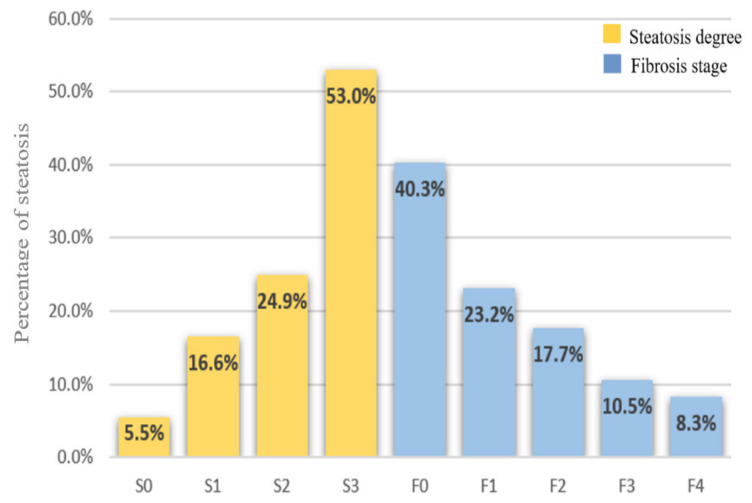
Proportion of patients according to steatosis degrees and fibrosis stages.

**Figure 2:**
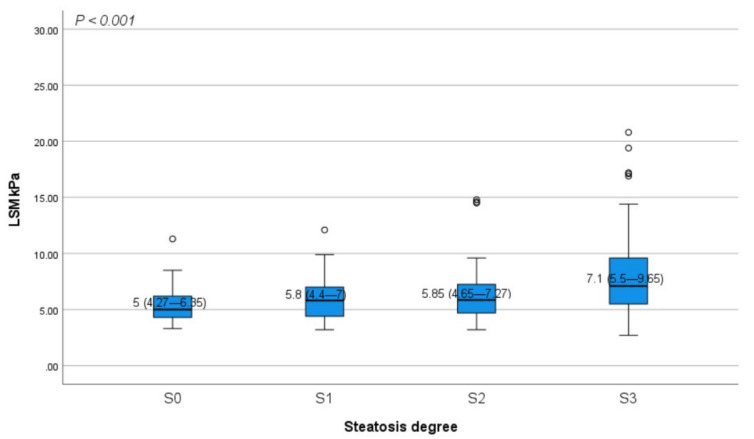
Distribution of LSM values according to steatosis degrees. The bottom and the top of each box represent the 25th and 75th percentiles, while the lines through the box indicate the median. The error bars indicate the 10th and 90th percentiles, excluding outliers (black circles). LSM, liver stiffness measurement.

The distribution of patients in S0, S1, S2, and S3 steatosis degrees based on CAP score was 10 (5.5%), 30 (16.6%), 45 (24.9%), and 96 (53%), as shown in Figure 1, with a median CAP value for each degree of 212 (179–226), 245 (241–247), 273.5 (264–283), and 333 dB/m (312–357), respectively. According to steatosis grades, the median value of LSM was 5 kPa (4.27–6.35) in patients without steatosis (S0), 5.8 kPa (4.4–7) in mild steatosis (S1), 5.85 kPa (4.65–7.27) in moderate steatosis (S2), and 7.1 kPa (5.5–9.65) in severe steatosis (S3), with an increased LSM value among steatosis degrees (*p* < 0.001) (Figure 2). From all patients included in the final analysis, 34 (18.8%) had advanced fibrosis (≥F3) with a median LSM value of 11.7 kPa (9.9–16.9). The LSM distribution according to METAVIR fibrosis scale was 73 (40.3%), 42 (23.2%), 32 (17.7%), 19 (10.5%), and 15 (8.3%) for F0, F1, F2, F3, and F4 grade, respectively, with a median for LSM score for each degree of 4.4 (3.8–5.1), 6.2 (5.8–6.2), 7.9 (7.45–8.5), 10.1 (9.7–11.05), and 17.1 kPa (14.55–21.85) (Figure 1). Among fibrosis stages, the median CAP value was 270 (232–321), 290 (242.5–342.5), 312 (272.5–336.5), 321 (263–348), and 314 dB/m (278–373).

### 3.3. Factors Associated with CAP and LSM

We performed a univariate linear regression analysis in order to identify risk factors associated with CAP and LSM, after which only those with a significant *p* value were included in multivariate regression analysis (Table 2). Multivariate analysis showed that BMI (β = 0.339, *p* < 0.001), fasting plasma glucose (β = 0.173, *p* = 0.046), triglycerides (β = 0.192, *p* = 0.043), LDL-c (β = 0.155, *p* = 0.008), and serum uric acid (β = 0.192, *p* = 0.003) were independent risk factors associated with CAP score in all patients. Age did not have a significant value in the multivariate analysis but remained strongly associated with the CAP value (β = 0.144, *p* = 0.053) in the univariate analysis. Furthermore, a lower platelet count (β = −0.168, *p* = 0.033) and albumin level (β = −0.295, *p* < 0.001) were also related to a higher LSM value, while serum uric acid (β = 0.339, *p* = 0.028) and AFP (β = 0.161, *p* = 0.033) levels were independently associated with an increased LSM score. Overall, patients with increased LSM values were older (59.6 ± 7.49 vs. 57.4 ± 8.12 years) than those with decreased LSM values, although age was not associated with the LSM value in the univariate analysis (β = 0.046, *p* = 0.537). In addition, we noticed that BMI, hypertension, and serum uric acid were associated with both CAP and LSM scores in the univariate analysis, while serum uric acid remained an independent risk factor in the multivariate linear regression analysis, associated with increased CAP and LSM values as well.

### 3.4. Correlation between Surrogate Serum Fibrosis Markers, LSM, and CAP

**Figure 3:**
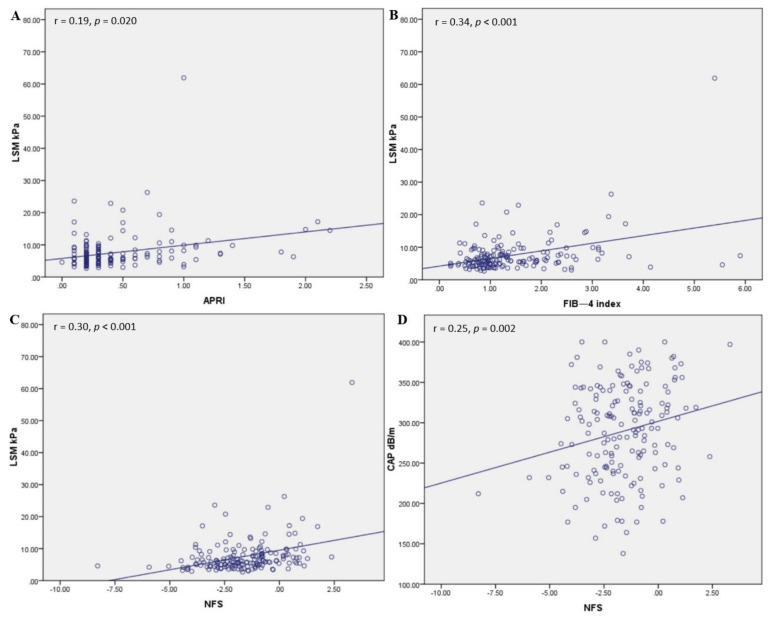
(**A**) Correlation between LSM and APRI, (**B**) FIB—4 index, and (**C**) NFS. (**D**) Correlation between CAP score and NFS.

For the overall cohort we found a significant correlation between LSM values and APRI (r = 0.19, *p* = 0.020) (Figure 3A), FIB-4 index (r = 0.34, *p* < 0.001) (Figure 3B), and NFS (r = 0.30 *p* < 0.001) (Figure 3C). Regarding hepatic steatosis expressed by CAP score, only NFS (r = 0.25, *p* = 0.002) maintained a positive, significant correlation (Figure 3D), while APRI (r = 0.11, *p* = 0.181) and FIB-4 (r = 0.07, *p* = 0.399) did not. 

## 4. Discussion

NAFLD is the most common chronic liver disease of our century, reaching epidemic proportions, with approximately 25% of the global population affected [1]. NAFLD-related fibrosis is the strongest predictor of poor outcomes in these patients who are at high risk for developing cirrhosis and HCC [6]. Therefore, early detection of liver fibrosis among NAFLD patients is mandatory for risk stratification and prevention of liver-related morbidity and mortality. As there are a number of limitations of LB to consider, several non-invasive methods have been developed for the assessment of hepatic fibrosis and steatosis and are nowadays used worldwide in clinical practice [21,22,23].

In our study, a high percentage of patients (94.5%) were diagnosed with hepatic steatosis (CAP ≥237 dB/m). Among them, 16.6% had S1, 24.9% had S2, and 53% had S3, proportions similar to those reported in another recently published study [30]. Furthermore, in our group, the patients diagnosed with steatosis by CAP were older, more likely to associate hypertension and T2DM, with higher BMI, LSM, FIB-4 index, and NFS values than those without steatosis. Moreover, metabolic abnormalities including an increased value of fasting glucose, ferritin, CRP, serum uric acid, total cholesterol, LDL-c, triglycerides, and a low level of HDL-c were more frequent in patients with steatosis compared with those with a CAP score <237 dB/m. In addition, upon multivariate analysis, we found that BMI (β = 0.339, *p* < 0.001), fasting plasma glucose (β = 0.173, *p* = 0.046), triglycerides (β = 0.192, *p* = 0.043), LDL-c (β = 0.155, *p* = 0.008), and serum uric acid (β = 0.192, *p* = 0.003) were independent risk factors associated with an increased CAP score. Our results are similar to those reported by several studies. Thus, Kwok et al. evaluated 1918 patients using VCTE and CAP and reported that steatosis was associated with higher values of BMI, triglycerides, fasting plasma glucose, and ALT, and with a low HDL-c level, respectively [31]. A recent meta-analysis by Jarvis et al., including 22 unique studies, found that T2DM and a BMI >30 kg/m^2^ were linked with an increased risk of developing severe liver disease. Moreover, lipid abnormalities (low HDL-c, high triglycerides level) and hypertension were independently associated with a high risk of incident severe liver disease events [32]. 

The majority of patients in this study had no liver fibrosis (F0: 73, 40.3%) or presented only mild (F1: 42, 23.2%) fibrosis, while the proportion of patients with significant (F2: 32, 17.7%), advanced (F3: 19, 10.5%) fibrosis and cirrhosis (F4: 15, 8.3%) was lower. It should be noted that the LSM value increased significantly in parallel with the degree of steatosis (S0 to S3, *p* < 0.001), with a strong correlation between LSM and serum fibrosis markers such as APRI (r = 0.19, *p* = 0.020), FIB-4 index (r = 0.30 *p* < 0.001), and NFS (r = 0.30 *p* < 0.001). These findings are in accordance with those revealed by several other studies comparing the accuracy of FibroScan in detecting fibrosis and steatosis stages among histologically confirmed NAFLD patients, which reported a prevalence between 10.1% and 28% for F3 stage, and 7.1–18.8% in patients with cirrhosis [19,21,23,33,34,35]. Using the same non-invasive method as in our research for liver fibrosis and steatosis evaluation, Mansour et al. found 57.78%, 22.22%, 17.78%, and 2.2% of patients with F0, F1, F2, and F4 stages, while no patient was found with advanced fibrosis (F3) [36]. These results are in contradiction with ours, in what concerns the percentage of patients with advanced fibrosis and cirrhosis stages, possibly due to a smaller number of patients included (90 NAFLD patients) in Mansour’s study. Fallatah et al. reported a more alarming proportion of patients with cirrhosis stage (32.8%), in a recent cross-sectional study including 122 NAFLD patients examined using FibroScan^®^ [37]. A possible reason for this high prevalence of cirrhosis among NAFLD patients could be the demographic differences between the studied populations, considering the high incidence of MeS and T2DM among the Saudi population that favors the progression of liver fibrosis in NAFLD patients. 

On the other hand, we found by multivariate analysis a significant, negative association between platelet count (β = −0.168, *p* = 0.033), albumin level (β = −0.276, *p* < 0.001), and LSM value. These data are consistent with those from the current literature, showing that thrombocytopenia and hypoalbuminemia are negatively correlated with severe fibrosis and cirrhosis [37,38]. We also found that serum uric acid (β = 0.192, *p* = 0.028) and AFP (β = 0.161, *p* = 0.033) are independent risk factors associated with an increased LSM value. Uric acid is related to the severity of liver damage as it promotes hepatic stellate cells activation and, subsequently, fibrosis development, while it is independently associated with HCC and NASH also in patients with NAFLD [39,40,41]. In a meta-analysis by Jaruvongvanich et al. that included five studies with biopsy-proved NAFLD patients, the authors found a significant correlation between hyperuricemia and NAFLD. Furthermore, subjects with elevated levels of serum uric acid had a high NAFLD activity score and a higher degree of histological liver damage [41].

The main limitation of our study was the absence of LB in the assessment of liver steatosis and fibrosis using only VCTE and CAP. In addition, we did not use different cut-off values for M and XL probes for fibrosis and steatosis evaluation. However, the prospective design of the study outweighs these shortcomings.

## 5. Conclusions

In conclusion, our findings show an important liver fibrosis prevalence among patients with clinically suspected or previously diagnosed NAFLD by abdominal US. In addition, these results underline that the presence of MeS components is a risk factor associated with both steatosis and fibrosis. Therefore, the assessment of liver fibrosis and steatosis by VCTE and CAP should be carried out in all units with available facilities. Moreover, we believe that it is high time for a screening program to be developed for diagnosis and evaluation of NAFLD to reduce the risk of chronic liver disease progression.

## Figures and Tables

**Table 1:** Baseline characteristics of patients according to the presence of hepatic steatosis (≥S1).

	Overall Cohort	No Steatosis	Any Steatosis (≥S1)	*p*–Value
Patients characteristics	*n* = 181	*n*, (%) = 10 (5.5)	*n,* (%) = 171 (94.5)	
Gender (female), n (%)	96 (53)	4 (40)	92 (54)	0.184
Age, y	57.62 ± 11.8	51.08 ± 12.74	59.19 ± 11.05	<0.001
BMI (kg/m^2^)	29.48 ± 4.85	25.76 ± 4.30	30.37 ± 4.56	<0.001
BMI ≥ 30 kg/m^2^, n (%)	87 (48)	2 (20)	86 (50.3)	<0.001
Diabetes, n (%)	39 (21.5)	1 (10)	38 (25.9)	<0.001
Hypertension, n (%)	54 (29.8)	2 (20)	52 (30.4)	<0.001
Platelet count (G/L)	251.67 ± 81.18	256.42 ± 131.39	250.53 ± 64.23	0.798
ALT (IU/L)	40.2 ± 41.29	29.75 ± 19.34	42.72 ± 44.71	0.136
AST (IU/L)	31.77 ± 22.61	25.21 ± 11.15	33.35 ± 23.37	0.079
GGT (IU/L)	58.89 ± 67.91	41.64 ± 31.07	57.33 ± 68.03	0.237
ALP (IU/L)	80.70 ± 36.92	76.67 ± 30.65	79.37 ± 35.01	0.709
Total bilirubin (mg/dL)	0.70 ± 0.38	0.75 ± 0.39	0.69 ± 0.38	0.465
Albumin (g/dL)	4.56 ± 0.38	4.53 ± 0.44	4.57 ± 0.36	0.559
Creatinine (mg/dL)	0.83 ± 0.13	0.814 ± 0.13	0.834 ± 0.13	0.505
Urea (mg/dL)	36.56 ± 10.81	34.40 ± 37.08	37.08 ± 11.22	0.188
Fasting glucose (mg/dL)	111.37 ± 43.77	96.37 ± 18.86	114.96 ± 47.19	0.024
Ferritin (ng/mL)	146.12 ± 114.98	111.54 ± 81	154.41 ± 120.47	0.047
CRP (mg/dL)	0.62 ± 2.27	1.38 ± 5.06	0.437 ± 0.482	0.048
Total cholesterol (mg/dL)	211.68 ± 55.04	191.61 ± 67.19	216.52 ± 50.85	0.031
Triglycerides (mg/dL)	148 ± 98.78	124.03 ± 113.49	153.78 ± 94.53	0.153
LDL-c (mg/dL)	125.34 ± 47.02	104.5 ± 45.59	130.37 ± 46.15	0.009
HDL-c (mg/dL)	45.06 ± 13.90	51.5 ± 13.47	43.5 ± 13.6	0.006
Serum uric acid (mg/dL)	5.21 ± 1.63	4.25 ±1.7	5.44 ± 1.53	<0.001
Alpha-fetoprotein (ng/mL)	3.84 ± 1.73	3.73 ± 1.13	3.87 ± 1.85	0.703
APRI	0.38 ± 0.31	0.34 ± 0.22	0.39 ± 0.33	0.288
FIB-4	1.36 ± 0.96	1.18 ± 0.33	1.41 ± 0.92	0.027
NFS	−1.61 ± 1.61	−2.37 ± 1.96	−1.44 ± 1.48	0.007
LSM (kPa)	6.1 (4.8–8.3)	5 (4.27–6.35)	6.3 (5.1–8.85)	0.049
CAP dB/m	293 (245.5–339)	212(178.75–226.5)	312 (273.5–344)	<0.001

BMI, body mass index; ALT, alanine aminotransferase; AST, aspartate aminotransferase; GGT, gamma-glutamyl transferase; ALP, alkaline phosphatase; LDL-c, low-density lipoprotein cholesterol; HDL-c, high-density lipoprotein cholesterol; LSM, liver stiffness measurement; CAP, controlled attenuation parameter; CRP, c-reactive protein, APRI, aspartate aminotransferase to platelet ratio; FIB-4, fibrosis-4 index; NFS, NAFLD fibrosis score.

**Table 2:** Factors associated with controlled attenuation parameter values and liver stiffness measurement using univariate and multivariate linear regression analysis.

	CAP				LSM			
Variable	Univariate		Multivariate		Univariate Multivariate			
	β	*p*	β	*P*	β	*P*	β	*P*
Gender	0.010	0.889			0.106	0.155		
Age, y	0.167	0.025	0.144	0.053	0.046	0.537		
BMI (kg/m^2^)	0.542	<0.001	0.339	<0.001	0.244	0.002	0.094	0.299
Diabetes	0.233	<0.001	0.020	0.790	0.141	0.058		
Hypertension	0.382	<0.001	0.132	0.084	0.231	0.002	0.093	0.257
Platelet (G/L)	−0.008	0.913			−0.258	<0.001	−0.168	0.033
ALT (IU/L)	0.170	0.041	0.132	0.361	−0.013	0.874		
AST (IU/L)	0.172	0.039	0.115	0.889	0.038	0.650		
GGT (IU/L)	0.124	0.137			0.197	0.018	0.024	0.760
ALP (IU/L)	0.069	0.413			0.170	0.042	0.077	0.325
TB (mg/dL)	−0.086	0.304			−0.044	0.600		
Albumin (g/dL)	0.079	0.345			−0.295	<0.001	−0.276	<0.001
Creatinine (mg/dL)	0.044	0.600			0.057	0.494		
Urea (mg/dL)	0.150	0.044	−0.005	0.938	0.095	0.204		
Fasting plasma glucose (mg/dL)	0.160	0.031	0.173	0.046	0.045	0.551		
Ferritin (ng/mL)	0.092	0.218			0.015	0.845		
CRP (mg/dL)	−0.112	0.182			−0.014	0.867		
TC (mg/dL)	0.177	0.034	0.048	0.525	−0.102	0.225		
Triglycerides (mg/dL)	0.267	0.001	0.192	0.043	0.008	0.924		
LDL-c (mg/dL)	0.370	<0.001	0.155	0.008	0.127	0.131		
HDL-c (mg/dL)	−0.254	0.002	−0.008	0.913	−0.118	0.157		
Serum uric acid (mg/dL)	0.482	<0.001	0.244	0.003	0.339	<0.001	0.192	0.028
AFP (ng/mL)	0.075	0.369			0.211	0.011	0.161	0.033
LSM (kPa)	0.226	0.002	0.038	0.591				
CAP (dB/m)					0.226	0.002	0.060	0.540

BMI, body mass index; ALT, alanine aminotransferase; AST, aspartate aminotransferase; GGT, gamma-glutamyl transferase; ALP, alkaline phosphatase; LDL-c, low-density lipoprotein cholesterol; HDL-c, high-density lipoprotein cholesterol; LSM, liver stiffness measurement; CAP, controlled attenuation parameter; CRP, c-reactive protein, AFP, alpha-fetoprotein; FG, fasting plasma glucose; TB, total bilirubin.

## Data Availability

The data presented in this study are available on request from the corresponding author. The data are not publicly available because they are the property of the Institute of Gastroenterology and Hepatology, Iasi, Romania.
